# MALDI-TOF mass spectrometry for sub-typing of *Streptococcus pneumoniae*

**DOI:** 10.1186/s12866-020-02052-7

**Published:** 2020-12-01

**Authors:** Sivkheng Kann, Sena Sao, Chanleakhena Phoeung, Youlet By, Juliet Bryant, Florence Komurian-Pradel, Vonthanak Saphonn, Monidarin Chou, Paul Turner

**Affiliations:** 1 Rodolphe Mérieux Laboratory, University of Health Sciences, Phnom Penh, Cambodia; 2 Cambodia Oxford Medical Research Unit, Angkor Hospital for Children, PO Box 50, Siem Reap, Cambodia; 3 Fondation Mérieux, Phnom Penh, Cambodia; 4 Fondation Mérieux and Centre International de Recherche en Infectiologie (CIRI), INSERM, Lyon, France; 5 University of Health Sciences, Phnom Penh, Cambodia; 6 Centre for Tropical Medicine and Global Health, Nuffield Department of Clinical Medicine, University of Oxford, Oxford, UK

**Keywords:** *Streptococcus pneumoniae*, Serotype, Genotype, MALDI-TOF, Mass spectrometry

## Abstract

**Background:**

Serotyping of *Streptococcus pneumoniae* is important for monitoring of vaccine impact. Unfortunately, conventional and molecular serotyping is expensive and technically demanding. This study aimed to determine the ability of matrix-assisted laser desorption-ionisation time-of-flight (MALDI-TOF) mass spectrometry to discriminate between pneumococcal serotypes and genotypes (defined by global pneumococcal sequence cluster, GPSC). In this study, MALDI-TOF mass spectra were generated for a diverse panel of whole genome sequenced pneumococcal isolates using the bioMerieux VITEK MS in clinical diagnostic (IVD) mode. Discriminatory mass peaks were identified and hierarchical clustering was performed to visually assess discriminatory ability. Random forest and classification and regression tree (CART) algorithms were used to formally determine how well serotypes and genotypes were identified by MALDI-TOF mass spectrum.

**Results:**

One hundred and ninety-nine pneumococci, comprising 16 serotypes and non-typeable isolates from 46 GPSC, were analysed. In the primary experiment, hierarchical clustering revealed poor congruence between MALDI-TOF mass spectrum and serotype. The correct serotype was identified from MALDI-TOF mass spectrum in just 14.6% (random forest) or 35.4% (CART) of 130 isolates. Restricting the dataset to the nine dominant GPSC (61 isolates / 13 serotypes), discriminatory ability improved slightly: the correct serotype was identified in 21.3% (random forest) and 41.0% (CART). Finally, analysis of 69 isolates of three dominant serotype-genotype pairs (6B-GPSC1, 19F-GPSC23, 23F-GPSC624) resulted in the correct serotype identification in 81.1% (random forest) and 94.2% (CART) of isolates.

**Conclusions:**

This work suggests that MALDI-TOF is not a useful technique for determination of pneumococcal serotype. MALDI-TOF mass spectra appear more associated with isolate genotype, which may still have utility for future pneumococcal surveillance activities.

**Supplementary Information:**

The online version contains supplementary material available at 10.1186/s12866-020-02052-7.

## Background

*Streptococcus pneumoniae,* a globally important pathogenic bacterium [1], consists of at least 100 distinct capsular serotypes [2]. Serotype-based surveillance of pneumococcal populations remains important since the polysaccharide capsule is a major antigen and the basis of current pneumococcal conjugate vaccines (PCV). Temporal changes to the serotypes associated with colonisation and disease may necessitate alterations to vaccine composition [3]. Traditional capsular typing by the Quellung reaction is both expensive and time-consuming. Deduction of serotype is possible by molecular techniques, including polymerase chain reaction (PCR) [4], microarray [5], and whole genome sequencing (WGS) [6]. However, these techniques are often technically demanding and/or not affordable in many settings. Although still largely unaffordable in resource-limited settings, bacterial identification is being done increasingly by matrix-assisted laser desorption-ionisation time-of-flight (MALDI-TOF) mass spectrometry, in both clinical and research laboratories [7]. To improve pneumococcal identification, a combined bile solubility test – MALDI-TOF assay has been developed to separate *S. pneumoniae* from the closely related *S. mitis* group of organisms [8]. Although MALDI-TOF mass spectra are derived from peptides / proteins, several studies have assessed the potential of MALDI-TOF for identification of pneumococcal serotypes. Encouragingly, in two of these, MALDI-TOF mass spectra clustering identified common pneumococcal serotypes fairly well [9, 10]. However, the most recently published study yielded considerably less optimistic results [11].

In view of the conflicting published data, we set out to determine whether MALDI-TOF mass spectra of pneumococci cluster consistently by serotype. A further aim was to explore whether any potential MALDI-TOF mass spectrum – serotype correlations were independent of underlying isolate genotype.

## Results

### Pneumococcal serotypes and genotypes

A total of 199 *S. pneumoniae* isolates were included in the study. PCR serotype results were concordant with phenotypic-WGS result in 198/199 to the level determined by PCR specificity (for some, this was to just the serogroup). One isolate was identified as serotype 6C by PCR but was non-typeable (NT) by both WGS and phenotypic methods. In the subsequent analyses, this isolate was referred to as NT.

To determine overall MALDI-TOF mass spectra clustering by serotype (objective 1), 130 of the isolates were examined. This selection included 16 serotypes plus non-typeable isolates (5 – 22 isolates per serotype). After further analysis of WGS data, a finalised genotype (sequence type [ST] / global pneumococcal sequence cluster [GPSC]) could be determined for all but three NT isolates: 46 GPSC were identified (Table 1).

**Table 1:** Characteristics of 130 pneumococcal isolates used to determine overall serotype discriminatory ability of MALDI-TOF

Serotype	Isolates	GPSC (n)	MLST (n)^a^
**1**	**5**	2 (5)	217 (5)
**6A**	**9**	6 (1), 23 (1) 47 (1), 87 (1), 623 (3), 625 (2)	27 ~ (1), 95 (1), 166 (1), 315 (1), 5421 (1), 5421 ~ (1), 5963 (1), 6334 ~ (1)
**6B**	**22**	9 (1), 23 (7), 37 (1), 40 (1), 47 (3), 87 (1), 180 (5), 623 (1), 625 (1), 796 (1)	27 ~ (1), 63 (1), 90 (1), 90 ~ (1), 95 (1), 95 ~ (1), 101 ~ (1), 292 ~ (1), 315 (1), 357 ~ (1), 2528 ~ (1), 2642 ~ (1), 2782 ~ (1), 3234 ~ (1), 3246 (1), 3815 ~ (1), 4966 (1), 5421 ~ (1), 5456 ~ (1), 6046 (1), 6917 (1), 10,060 (1)
**11A**	**7**	6 (2), 9 (1), 22 (1), 23 (1), 73 (1), 626 (1)	95 (1), 99 (1), 166 (1), 166 ~ (1), 782 (1), 4440 ~ (1), 5681 ~ (1)
**13**	**7**	134 (3), 320 (2), 295 (1), 637 (1)	734 ~ (1), 1260 (1), 4389 ~ (1), 5210 ~ (1), 7539 ~ (1), 10,374 (1), 10,374 ~ (1)
**14**	**7**	9 (6), 28 (1)	63 (1), 63 ~ (1), 782 (1), 782 ~ (2), 1914 ~ (1), 4396 (1)
**15A**	**5**	9 (2), 69 (2), 222 (1)	3058 ~ (2), 3130 ~ (1), 4561 (1), 8152 (1)
**15B**	**5**	10 (1), 48 (3), 230 (1)	230 ~ (1), 1961 (1), 1961 ~ (2), 10,086 ~ (1)
**15C**	**5**	48 (5)	1961 (2), 1961 ~ (3)
**18C**	**5**	16 (3), 142 (2)	3180 ~ (3), 3594 (2)
**19A**	**7**	1 (4), 10 (1), 23 (1), 798 (1)	95 (1), 230 (1), 236 ~ (1), 320 (1), 320 ~ (1), 2267 (1), 4927 (1)
**19F**	**5**	1 (4), 624 (1)	236 (1), 236 ~ (1), 2694 ~ (1),7758 ~ (1), 9050 (1)
**23A**	**5**	5 (1), 40 (1), 626 (3)	388 ~ (1), 5681 (1), 5681 ~ (1), 8011 ~ (2)
**23F**	**12**	1 (1), 10 (1), 14 (1), 16 (1), 20 (1), 222 (2), 624 (4), 626 (1)	81 (1), 230 (1), 243 ~ (1), 271 ~ (1), 4639 (1), 5681 ~ (1), 8152 (1), 8152 ~ (1), 9050 (1), 9050 ~ (1), 10,637 (1), 10,637 ~ (1)
**34**	**5**	45 (5)	1439 (4), 1439 ~ (1)
**35B**	**5**	13 (2), 59 (1), 147 (1), 671 (1)	473 (1), 473 ~ (1), 558 (1), 12,641 ~ (1), 5532 ~ (1)
**NT**	**14**	60 (2), 319 (1), 397 (1), 628 (4), 795 (1), 800 (1), 805 (1), NA^b^ (3)	41 ~ (1), 448 (1), 725 ~ (1), 954 ~ (1), 1993 ~ (1), 2337 ~ (1), 6666 ~ (1), 7022 ~ (1), 8966 (1), 10,383 ~ (1), 10,500 (1), NA^b^ (3)

^a^ "~" following a ST denotes a single-locus variant of that ST (i.e. 6/7 loci match). ^b^ Not available

**Fig. 1:**
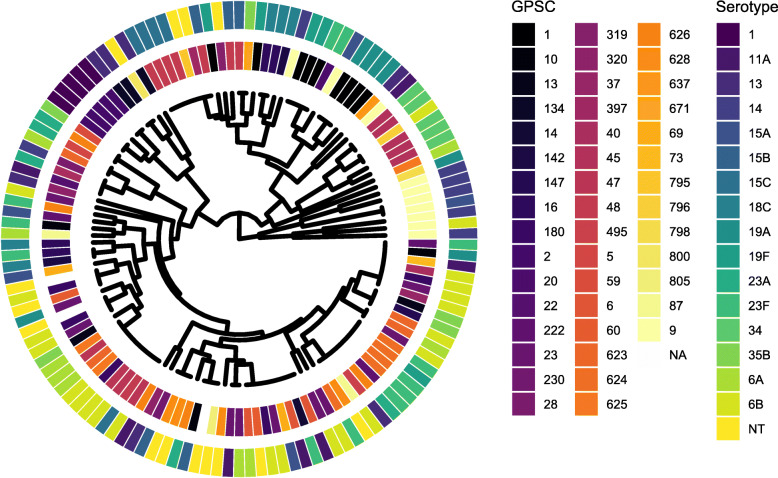
A cluster dendrogram of serotype-organised MALDI-TOF mass spectrum data for 16 serotypes + non-typeable (NT) isolates. The isolate selection includes 130 pneumococcal isolates from 46 global pneumococcal sequence clusters (GPSC). The inner metadata ring denotes GPSC and the outer ring serotype

**Fig. 2:**
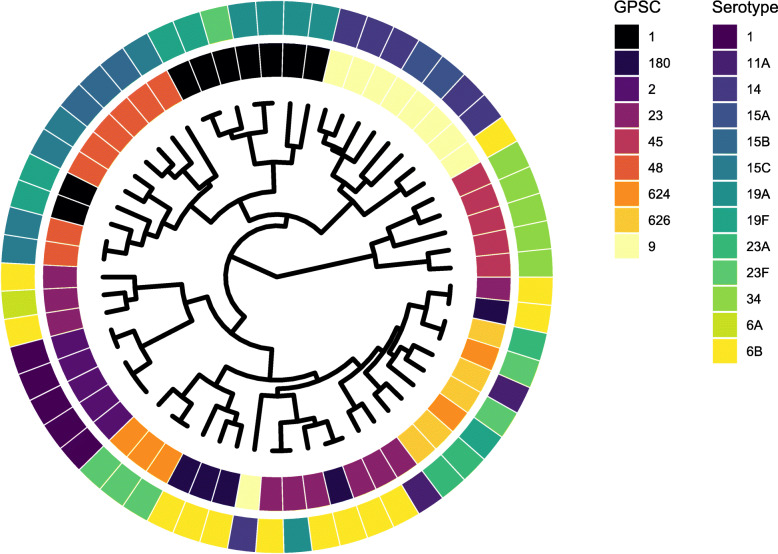
A cluster dendrogram of serotype-organised MALDI-TOF mass spectrum data including only genotypes with ≥ 5 isolates. The isolate selection includes 61 isolates comprising 13 serotypes and nine global pneumococcal sequence clusters (GPSC). The inner metadata ring denotes GPSC and the outer ring serotype

From 785 matched peaks, 16 peaks were found to discriminate between serotypes (false discovery rate [FDR] q < 0.05; Table 2). Hierarchical clustering, on the basis of these peaks, identified four major clusters (Fig. 1). Most serotypes (13/16; 81.3%) appeared in > 1 cluster, with just serotypes 1, 23A, and 34 confined to a single cluster. Serotypes 1 and 34 were the least genotypically diverse, being represented by a single GPSC each. The MALDI-TOF mass spectra from the same 130 isolates was re-analysed following reorganisation of the dataset by genotype (GPSC) rather than serotype. This identified 27 discriminatory peaks and three major clusters. Only 3/46 (6.5%) GPSCs were spread across more than one cluster (Additional File 1). Classification of isolate mass spectrum data by random forest or classification and regression tree (CART) algorithms was sub-optimal. The random forest approach identified the correct serotype just in 14.6%, and GPSC in 17.7%, of isolates; CART correctly identified serotype in 35.4% and GPSC in 27.7% of isolates (Additional File 2). Restricting the dataset to the nine dominant GPSC, those comprising of at least five isolates (total 61 isolates, 13 serotypes; 535 matched peaks), discriminatory ability improved to some degree (Fig. 2 [serotype-organised data; 16 discriminatory peaks] and Additional File 3 [genotype-organised data; 34 discriminatory peaks]). With serotype-organised data, correct serotype was identified in 21.3% (random forest) and 41.0% (CART) instances. Using genotype-organised data, correct GPSC was identified in 45.9% (random forest) and 77.0% (CART) instances (Additional File 4).

**Table 2:** Discriminant peak list derived from 130 pneumococcal isolates, comprising 16 serotypes + non-typeable (NT) isolates

Peak (m/z)	FDR^a^ q-value	Serotype																
		1	6A	6B	11A	13	14	15A	15B	15C	18C	19F	19A	23F	23A	34	35B	NT
3384.45	0.02093	1.00	0.00	0.14	0.14	0.29	0.29	0.00	0.00	0.00	0.20	0.00	0.00	0.08	0.00	0.20	0.20	0.00
3529.99	0.00714	0.40	0.33	0.14	0.71	0.00	0.14	0.20	0.80	0.80	0.00	0.20	0.71	0.08	0.60	0.40	0.00	0.64
3538.70	0.03434	0.20	0.11	0.05	0.00	0.57	0.43	0.20	0.00	0.00	0.80	0.00	0.14	0.08	0.00	0.00	0.00	0.14
4018.95	0.00000	0.00	0.11	0.09	0.14	0.00	0.57	0.20	0.00	0.00	0.00	0.00	0.14	0.00	0.80	0.00	0.60	0.00
4198.34	0.00714	1.00	0.44	0.64	0.86	0.57	0.14	0.20	0.20	0.00	0.00	0.20	0.29	0.58	1.00	0.00	0.60	0.50
4212.94	0.00000	0.00	0.00	0.05	0.14	0.14	0.43	0.20	0.60	0.80	0.60	0.80	0.71	0.17	0.00	1.00	0.20	0.21
4774.71	0.00000	0.00	0.11	0.05	0.00	0.00	0.00	0.00	0.00	0.00	0.00	0.00	0.00	0.00	0.00	1.00	0.00	0.00
4973.01	0.00000	1.00	0.11	0.09	0.14	0.71	0.29	0.40	0.40	0.00	0.00	1.00	0.71	0.75	0.60	0.00	0.20	0.43
5062.44	0.02093	0.00	0.00	0.05	0.00	0.00	0.57	0.20	0.00	0.00	0.00	0.00	0.00	0.00	0.00	0.00	0.00	0.00
6509.37	0.00000	1.00	0.33	0.14	0.14	0.57	0.43	0.60	0.00	0.00	0.00	0.40	0.86	0.17	0.20	0.40	0.00	0.07
7061.16	0.00000	1.00	0.00	0.00	0.00	0.14	0.00	0.00	0.00	0.00	0.00	0.00	0.14	0.00	0.00	0.00	0.00	0.07
8039.25	0.01963	0.00	0.11	0.00	0.14	0.00	0.57	0.00	0.00	0.00	0.00	0.00	0.14	0.00	0.60	0.00	0.20	0.00
8396.71	0.00000	1.00	0.67	0.55	0.71	0.71	0.14	0.40	0.40	0.00	0.00	0.20	0.14	0.67	1.00	0.00	0.60	0.71
8426.29	0.00000	0.00	0.00	0.05	0.00	0.14	0.57	0.40	0.40	0.80	1.00	0.80	0.71	0.25	0.00	1.00	0.20	0.21
9548.41	0.00000	0.00	0.11	0.05	0.00	0.00	0.00	0.00	0.00	0.00	0.00	0.00	0.00	0.00	0.00	1.00	0.00	0.07
10,121.74	0.02093	0.00	0.00	0.05	0.00	0.00	0.57	0.20	0.00	0.00	0.00	0.00	0.00	0.00	0.00	0.00	0.00	0.00

The number in each cell summarises the proportion of isolates of the serotype with the corresponding mass peak. ^a^False discovery rate.

**Fig. 3:**
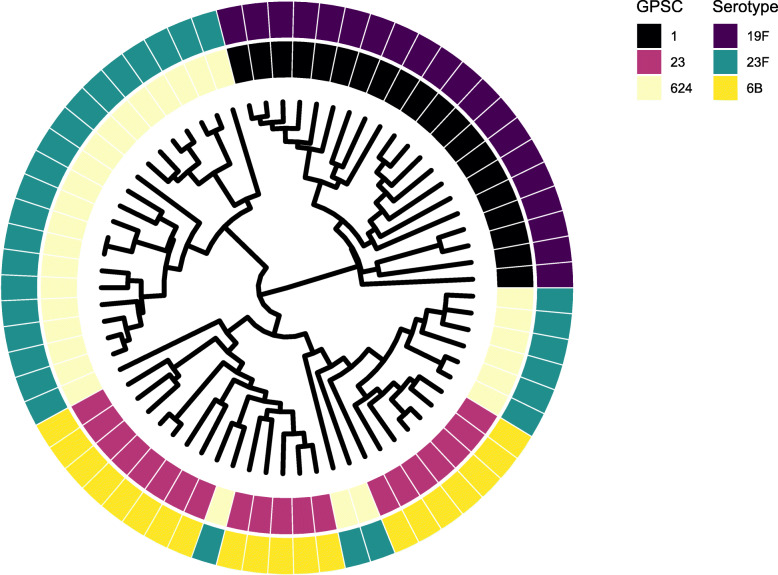
A cluster dendrogram of serotype-organised MALDI-TOF mass spectrum data for the three dominant pneumococcal serotype-genotype pairs. The isolate selection comprises 69 pneumococcal isolates. The inner metadata ring denotes global pneumococcal sequence type (GPSC) and the outer ring serotype

For assessment of stability of MALDI-TOF mass spectra within a serotype-genotype (objective 2), the remaining 69 isolates were examined. This selection included 20 isolates from the major ST of serotypes 6B (ST95 [GPSC1]) and 19F (ST236 [GPSC23]). For serotype 23F, 29 isolates from the two dominant ST were included (ST9050 and ST10637 [both GPSC624]). Thirty-two discriminatory peaks were identified from a total of 562 peaks (FDR q < 0.05, Additional File 5). Four clusters were identified (Fig. 3 [serotype-organised data] and Additional File 6 [genotype-organised data]). For serotype-organised data, the correct serotype was identified in 81.1% (random forest) and 94.2% (CART) of isolates. For genotype-organised data, the correct GPSC was classified in 76.8% (random forest) and 97.1% (CART) of isolates (Additional File 7).

## Discussion

This study failed to identify consistent clustering of MALDI-TOF mass spectrum by serotype in a collection of well-characterised pneumococcal isolates with diverse genotypes. Reducing the number of genotypes within the dataset analysed improved classification of serotype by MALDI-TOF mass spectrum. Inclusion of just three serotypes, and one genotype per serotype, resulted in correct serotype classification in > 90% of isolates. Overall, this suggests that MALDI-TOF mass spectrum clustering within *S. pneumoniae* is driven by underlying genotype.

Our results contrast slightly with two previous studies which both found that, with careful isolate selection and optimisation of peak lists, MALDI-TOF could discriminate between several common serotypes. From a Japanese collection of 407 isolates from 10 major serotypes, a ClinProTools-developed classification algorithm correctly identified serotypes in 84.0% of isolates (9). Although multiple genotypes were included for each serotype, a dominant ST could be identified in several of them. The authors concluded that further work to determine the interaction between pneumococcal genotype and MALDI-TOF mass spectrum would be helpful. Analysis of 416 Brazilian isolates from six serotypes identified 10 major clusters by visualising a neighbour joining tree based on Pearson’s coefficient [10]. Whilst visually serotypes did cluster fairly well, it was notable that all serotypes were identified in > 1 cluster. Importantly, genotyping data were not available in this study.

The major strength of the study is that it included whole genome sequenced isolates where serotype had been verified by both molecular and phenotypic methods. The MALDI-TOF work was performed using the machine in clinical diagnostic / IVD mode, i.e. as the data would be generated in routine clinical microbiology laboratory. The study utilised fully open-source and freely available analytic tools [12, 13], in contrast to previous analyses of pneumococcal MALDI-TOF data [9, 10]. These tools permitted more exploratory analyses and visualisation of the data, rather than being constrained to machine-learning algorithms packaged with the machine software (often Support Vector Machine [14]). However, there are several limitations to note. The sample size was small, resulting in a small number of isolates for some serotypes. It would have been helpful to have a larger number of isolates of each serotype-genotype pair. To minimise minor variations in mass spectra, inclusion of multiple mass spectra per isolate could have been done. However, this added analytic complexity would not have been reflective of normal diagnostic workflow. To mitigate this limitation, the final dataset included only those isolates where the automated analysis of mass spectrum data had indicated acceptable identification at the species level. In this context, isolates with an initially unacceptable species identification likely represented technical errors in the laboratory (i.e. poor slide preparation). Thus, isolates not meeting this criterion were repeated to obtain acceptable MALDI-TOF mass spectra. It is also encouraging to note that several of the discriminatory masses in Table 1 (e.g. 5062.44 / 10,121.74) display similar peak proportions within a serotype, indirectly demonstrating spectrum reproducibility. Finally, as has been noted previously, it would have been optimal to have included isolates from more than one geographic location and to have performed external validation of the random forest / CART models. This latter point was highlighted as a major roadblock to progress in a recent systematic review [14]. Despite these limitations, our findings and conclusions are similar to those of Ercibengoa et al [11]. This carefully conducted study of 60 isolates of four common pneumococcal serotypes failed to confirm presence of either novel or previously established discriminatory MALDI-TOF peaks. The study team speculate that proteins associated with capsular polysaccharide synthesis are likely to be outside of the MALDI-TOF detection range. They noted also that genotype differences may render cross-site validation and use of external discriminatory peaks challenging.

The pneumococcal capsule synthesis locus (*cps*) is remarkably complex, with considerable diversity within the key enzyme classes [15, 16]. The relationship between *cps* locus gene content and immunologically determined serotype is not always straightforward. In an analysis of the *cps* loci of 88 serotypes with capsules synthesised by the Wzy-dependent pathway, eight major clusters and 21 sub-clusters were identified [17]. In the majority of cases, members of the same serogroup were co-located in the same cluster, however there were several examples where this relationship broke down. Taken together, all of these findings should perhaps temper enthusiasm for further attempts at MALDI-TOF-based serotyping of *S. pneumoniae.* However, if genotypic differences in pneumococcal MALDI-TOF mass spectra are found to be consistent between sites and with sequence-based genotype data, then MALDI-TOF could still have a potential role in future pneumococcal surveillance. Indeed, there is precedent for this, as it has been shown already that variations in ribosomal protein mass peaks correlated with clonal complex in *Neisseria meningitidis* [18]*.* With the rapid proliferation of pneumococcal sequencing globally, the availability of ribosomal protein sequence data from RiboDB [19], and the increasing use of MALDI-TOF for primary identification of isolates that are submitted for such sequencing, this should be amenable to exploration at scale.

## Conclusions

Identification of pneumococcal serotype by MALDI-TOF is not reliable. MALDI-TOF mass spectra appear more associated with underlying genotype. Further work is warranted to determine the robustness of pneumococcal genotype identification by MALDI-TOF.

## Methods

### Bacterial isolate selection

Pneumococcal isolates that had been characterised during pre- and post-PCV pneumococcal colonisation and disease studies of children attending for care at Angkor Hospital for Children in Cambodia were selected for further study [20, 21]. Isolates were selected for inclusion using the following criteria: (a) submitted for sequencing as part of the on-going Global Pneumococcal Sequencing project [22], and had passed initial WGS quality control (QC) checks with availability of preliminary in-silico MLST genotype; (b) WGS-derived and phenotypic serotype were congruent, including NT pneumococci as a “serotype”; (c) at least ten isolates per serotype.

To determine how well MALDI-TOF mass spectra clustered by serotype (objective 1), at least one isolate of each distinct ST identified within a serotype was included. If there were less than five different STs for a serotype, multiple isolates of the same serotype-ST were included to a total of five isolates. A total of 130 isolates were included in this work. To explore the stability of MALDI-TOF mass spectra within unique serotype-genotype pairs (objective 2), multiple isolates of the commonest ST, including single-locus variants if less than 20 isolates, were selected for serotypes 6B, 19F, and 23F, the dominant serotypes in the isolate collection. A total of 69 isolates were included in this work.

### Re-confirmation of serotype by multiplex PCR

As a further confirmation of serotype, DNA was extracted from re-cultured isolates using the QIAamp DNA Mini Kit (Qiagen, Hilden, Germany) and a multiplex real-time PCR was performed to detect 40 pneumococcal serotypes, as previously described [23]. This PCR also includes a primer-probe set for the *S. pneumoniae-*specific *lytA* (autolysin) gene, to confirm species identification.

### Characterisation by MALDI-TOF

The stored isolates were re-cultured overnight at 35-37 °C in 5% CO_2_ on 5% sheep blood agar (Oxoid, Basingstoke, UK; prepared in-house). Single colonies were mixed with matrix (alpha-cyano-4-hydroxycinnamic acid, CHCA) on disposable metallised slides and analysed using the VITEK MS MALDI-TOF system (bioMerieux, Marcy L’ Etoile, France), following manufacturer instructions. Particular care was taken with the preparation of colony-matrix spots, in order to optimise the generation of high-quality mass spectra. To be compatible with routine diagnostic laboratory workflow, a single mass spectrum was generated per isolate. The machine was run in diagnostic (IVD) mode, with automated measurement of proteins in the specific mass range of 2,000 – 20,000 Da. *Escherichia coli* ATCC 8739 was used for QC and calibration of each slide. Isolates were considered acceptable for analysis if the slide passed QC and the isolate was identified unambiguously as *S. pneumoniae* by the automated reporting system (bioMerieux Myla, Knowledge Base V3.2.0).

### Pneumococcal genotype assignment

Isolates were selected on the basis of MLST genotype. However, during the conduct of the study, the GPSC system was proposed as the optimal method for clustering of *S. pneumoniae* using WGS data [24]. GPSC were determined automatically for all isolates submitted to the GPS project and, thus, in the following analyses, GPSC have been used instead of ST to describe isolate genotypes. For clarity, both GPSC and ST are included in Table 1.

### Data analysis

MALDI-TOF mass spectra were exported as peak lists (.mzML files) from the VITEK-MS instrument: a single peak list per isolate. These were converted to text (.csv) files using the R statistical software V3.6.3 [25] and package “MALDIquant” [12]. Peak lists were stored in both serotype-isolate and genotype-isolate folder structures. These labelled peak lists were imported into MASS-Up, an open source MALDI-TOF analysis program, using the “load peak” command [13]. Inter-sample peak matching, by serotype or genotype, was performed using default settings (method - “forward”; tolerance type - “ppm”; tolerance - 300 ppm; reference type - “AVG”). Discriminant peaks were identified by using the biomarker discovery function. Discriminant peak lists (DPL) were generated by selecting peaks with a q-value of < 0.05 (Benjamini Hochberg false discovery rate). Hierarchical clustering was performed on these discriminatory peaks using the “hclust” function (method = “average”) in R, following generation of a distance matrix (Hamming distance, using the “hamming.distance” function of the “e1071” package [26]). Clusters were identified using the “mclust” and “NbClust” packages [27, 28]. For visual assessment of the relationship between MALDI-TOF mass spectra and serotype or genotype, circular dendrograms were visualized and annotated using the “ggtree” package [29]. Formal testing of discriminatory ability was done via the classification analysis functionality in MASS-Up, using all peak data. The “Random Forest” and “Classification and Regression Tree (CART)” algorithms were run using default settings and 10-fold cross validation.

## Supplementary Information

**Additional file 1.** A cluster dendrogram of genotype-organised MALDI-TOF mass spectrum data for 16 serotypes + non-typeable (NT) isolates.

**Additional file 2 **Characteristics of Random Forest and CART algorithms for serotype- and genotype-associated MALDI-TOF mass spectra from 130 *Streptococcus pneumoniae* isolates from 16 serotypes (plus non-typeable [NT] isolates) and 46 global pneumococcal sequence clusters (GPSC.

**Additional file 3.** A cluster dendrogram of genotype-organised MALDI-TOF mass spectrum data including only genotypes with ≥5 isolates.

**Additional file 4 **Characteristics of Random Forest and CART algorithms for serotype- and genotype-associated MALDI-TOF mass spectra from 61 *Streptococcus pneumoniae* isolates from nine dominant global pneumococcal sequence clusters (GPSC) comprised of 13 serotypes.

**Additional file 5.** Discriminant peak list / matrix derived from 69 pneumococcal isolates, comprising tree dominant serotype-genotype pairs.

**Additional file 6 **Characteristics of Random Forest and CART algorithms for serotype- and genotype-associated MALDI-TOF mass spectra from 69 *Streptococcus pneumoniae* isolates from three serotype - global pneumococcal sequence cluster (GPSC) pairs.

**Additional file 7.** A cluster dendrogram of serotype-organised MALDI-TOF mass spectrum data for the three dominant pneumococcal serotype-genotype pairs.

## Data Availability

The datasets used and/or analysed during the current study are available from the corresponding author on reasonable request.

## Abbreviations

CART: Classification and regression tree
CHCA: Alpha-cyano-4-hydroxycinnamic acid
DPL: Discriminant peak list
FDR: False discovery rate
GPS: Global pneumococcal sequencing study
GPSC: Global pneumococcal sequence cluster
IVD: In-vitro diagnostic
MALDI-TOF: Matrix-assisted laser desorption-ionisation time-of-flight
MLST: Multi-locus sequence typing
NT: Non-typeable
PCR: Polymerase chain reaction
PCV: Pneumococcal conjugate vaccine
QC: Quality control
ST: Sequence type
WGS: Whole genome sequence / sequencing
