# Supplementary material for: MALDI-TOF mass spectrometry for sub-typing of Streptococcus pneumoniae
Source: BMC Microbiol. 2020 Dec 1;20:367. doi: 10.1186/s12866-020-02052-7 (PMC7709296; doi:10.1186/s12866-020-02052-7)
Supplement: Supplementary file 1 — Additional file 1. A cluster dendrogram of genotype-organised MALDI-TOF mass spectrum data for 16 serotypes + non-typeable (NT) isolates. [file 12866_2020_2052_MOESM1_ESM.docx]

**A cluster dendrogram of genotype-organised MALDI-TOF mass spectrum data for 16 serotypes + non-typeable (NT) isolates**

The isolate selection includes 130 pneumococcal isolates from 46 global pneumococcal sequence clusters (GPSC). The inner metadata ring denotes GPSC and the outer ring serotype.

**
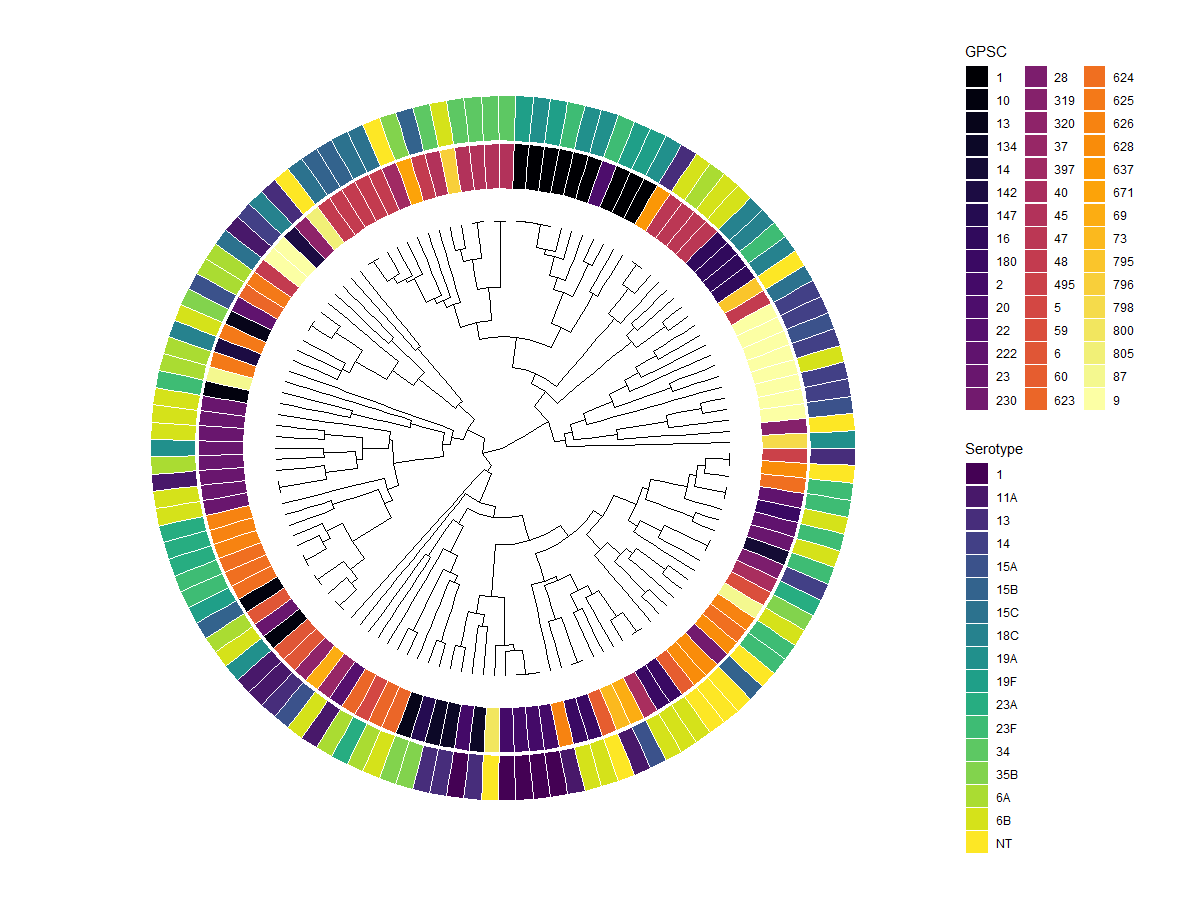
**
