# Supplementary material for: MALDI-TOF mass spectrometry for sub-typing of Streptococcus pneumoniae
Source: BMC Microbiol. 2020 Dec 1;20:367. doi: 10.1186/s12866-020-02052-7 (PMC7709296; doi:10.1186/s12866-020-02052-7)
Supplement: Supplementary file 2 — Additional file 2 Characteristics of Random Forest and CART algorithms for serotype- and genotype-associated MALDI-TOF mass spectra from 130 Streptococcus pneumoniae isolates from 16 serotypes (plus non-typeable [NT] isolates) and 46 global pneumococcal sequence clusters (GPSC. [file 12866_2020_2052_MOESM2_ESM.docx]

**Characteristics of Random Forest and CART algorithms for serotype- and genotype-associated MALDI-TOF mass spectra from 130 *Streptococcus pneumoniae* isolates from** **16 serotypes (plus non-typeable [NT] isolates) and 46 global pneumococcal sequence clusters (GPSC)**

| **Data organisation** | **Class** | **Random Forest** | | | | **CART** | | |
| --- | --- | --- | --- | --- | --- | --- | --- | --- |
|  |  | **TP Rate*** | **FP Rate†** | | **ROC‡ Area** | **TP Rate** | **FP Rate** | **ROC Area** |
| **Serotype** | 1 | 0.4 | 0.032 | | 0.96 | 1 | 0.016 | 0.99 |
|  | 6A | 0.333 | 0.058 | | 0.75 | 0.111 | 0.041 | 0.598 |
|  | 6B | 0.318 | 0.213 | | 0.61 | 0.727 | 0.241 | 0.741 |
|  | 11A | 0.143 | 0.033 | | 0.605 | 0 | 0.033 | 0.609 |
|  | 13 | 0 | 0.049 | | 0.654 | 0.143 | 0.016 | 0.527 |
|  | 14 | 0 | 0.016 | | 0.469 | 0.429 | 0.024 | 0.833 |
|  | 15A | 0 | 0.016 | | 0.562 | 0 | 0 | 0.39 |
|  | 15B | 0 | 0.008 | | 0.583 | 0 | 0 | 0.382 |
|  | 15C | 0 | 0 | | 0.694 | 0.4 | 0.04 | 0.718 |
|  | 18C | 0 | 0.024 | | 0.678 | 1 | 0.064 | 0.967 |
|  | 19F | 0 | 0.032 | | 0.667 | 0.2 | 0.016 | 0.744 |
|  | 19A | 0 | 0.041 | | 0.677 | 0.286 | 0.033 | 0.713 |
|  | 23F | 0.167 | 0.161 | | 0.554 | 0.5 | 0.144 | 0.785 |
|  | 23A | 0 | 0.024 | | 0.57 | 0 | 0.008 | 0.655 |
|  | 34 | 0.2 | 0.032 | | 0.733 | 0.8 | 0.016 | 0.887 |
|  | 35B | 0.2 | 0.048 | | 0.64 | 0 | 0.008 | 0.533 |
|  | NT | 0.143 | 0.155 | | 0.457 | 0 | 0.017 | 0.471 |
|  | **Weighted average** | **0.146** | **0.087** | | **0.619** | **0.354** | **0.071** | **0.675** |
| **Genotype** | GPSC1 | 0.444 | 0.149 | | 0.677 | 1 | 0.033 | 0.983 |
|  | GPSC2 | 0.4 | 0.056 | | 0.744 | 0.4 | 0.016 | 0.87 |
|  | GPSC5 | 0 | 0 | | 0.457 | 0 | 0 | 0.368 |
|  | GPSC6 | 0 | 0.008 | | 0.657 | 0 | 0 | 0.682 |
|  | GPSC9 | 0.222 | 0.033 | | 0.845 | 0.556 | 0 | 0.846 |
|  | GPSC10 | 0 | 0 | | 0.429 | 0 | 0.016 | 0.713 |
|  | GPSC13 | 0 | 0 | | 0.453 | 0 | 0 | 0.582 |
|  | GPSC14 | 0 | 0 | | 0.453 | 0 | 0 | 0.376 |
|  | GPSC16 | 0 | 0.016 | | 0.657 | 0 | 0.016 | 0.816 |
|  | GPSC20 | 0 | 0.008 | | 0.481 | 0 | 0 | 0.461 |
|  | GPSC22 | 0 | 0.008 | | 0.465 | 0 | 0 | 0.376 |
|  | GPSC23 | 0.6 | 0.133 | | 0.711 | 0.5 | 0.2 | 0.714 |
|  | GPSC28 | 0 | 0.008 | | 0.473 | 0 | 0 | 0.353 |
|  | GPSC37 | 0 | 0.008 | | 0.446 | 0 | 0 | 0.364 |
|  | GPSC40 | 0 | 0.031 | | 0.418 | 0 | 0.016 | 0.855 |
|  | GPSC45 | 0.4 | 0.016 | | 0.938 | 1 | 0.04 | 0.974 |
|  | GPSC47 | 0 | 0.079 | | 0.357 | 0.75 | 0.016 | 0.858 |
|  | GPSC48 | 0.5 | 0.098 | | 0.706 | 0.5 | 0.066 | 0.721 |
|  | GPSC59 | 0 | 0 | | 0.446 | 0 | 0 | 0.388 |
|  | GPSC60 | 0 | 0.016 | | 0.426 | 0 | 0 | 0.85 |
|  | GPSC69 | 0 | 0.016 | | 0.434 | 0 | 0 | 0.674 |
|  | GPSC73 | 0 | 0 | | 0.477 | 0 | 0 | 0.349 |
|  | GPSC87 | 0 | 0.008 | | 0.648 | 0 | 0 | 0.648 |
|  | GPSC134 | 0 | 0 | | 0.904 | 0.333 | 0.016 | 0.78 |
|  | GPSC142 | 0 | 0.016 | | 0.449 | 0 | 0 | 0.332 |
|  | GPSC147 | 0 | 0 | | 0.45 | 0 | 0 | 0.368 |
|  | GPSC180 | 0 | 0.008 | | 0.531 | 0 | 0.04 | 0.638 |
|  | GPSC222 | 0 | 0.016 | | 0.39 | 0 | 0.016 | 0.924 |
|  | GPSC230 | 0 | 0 | | 0.453 | 0 | 0 | 0.38 |
|  | GPSC319 | 0 | 0 | | 0.469 | 0 | 0 | 0.442 |
|  | GPSC320 | 0 | 0.016 | | 0.426 | 0 | 0 | 0.379 |
|  | GPSC397 | 0 | 0 | | 0.469 | 0 | 0 | 0.438 |
|  | GPSC495 | 0 | 0 | | 0.446 | 0 | 0 | 0.411 |
|  | GPSC623 | 0 | 0.024 | | 0.51 | 0 | 0.008 | 0.7 |
|  | GPSC624 | 0 | 0.065 | | 0.593 | 0.333 | 0.137 | 0.711 |
|  | GPSC625 | 0.333 | 0.008 | | 0.806 | 0 | 0.016 | 0.546 |
|  | GPSC626 | 0.25 | 0.008 | | 0.687 | 0 | 0.024 | 0.518 |
|  | GPSC628 | 0.25 | 0 | | 0.556 | 0 | 0.048 | 0.496 |
|  | GPSC637 | 0 | 0 | | 0.457 | 0 | 0 | 0.442 |
|  | GPSC671 | 0 | 0 | | 0.461 | 0 | 0 | 0.442 |
|  | GPSC795 | 0 | 0 | | 0.473 | 0 | 0 | 0.442 |
|  | GPSC796 | 0 | 0 | | 0.465 | 0 | 0 | 0.465 |
|  | GPSC798 | 0 | 0 | | 0.488 | 0 | 0 | 0.465 |
|  | GPSC800 | 0 | 0 | | 0.473 | 0 | 0 | 0.43 |
|  | GPSC805 | 0 | 0 | | 0.465 | 0 | 0 | 0.399 |
|  | GPSCNA | 0 | 0.016 | | 0.417 | 0 | 0.039 | 0.597 |
|  | **Weighted average** | **0.177** | | **0.042** | **0.611** | **0.277** | **0.038** | **0.691** |

*True positive, †False positive, ‡Receiver operating characteristic
