# Supplementary material for: MALDI-TOF mass spectrometry for sub-typing of Streptococcus pneumoniae
Source: BMC Microbiol. 2020 Dec 1;20:367. doi: 10.1186/s12866-020-02052-7 (PMC7709296; doi:10.1186/s12866-020-02052-7)
Supplement: Supplementary file 3 — Additional file 3. A cluster dendrogram of genotype-organised MALDI-TOF mass spectrum data including only genotypes with ≥5 isolates. [file 12866_2020_2052_MOESM3_ESM.docx]

**A cluster dendrogram of genotype-organised MALDI-TOF mass spectrum data including only genotypes with ≥5 isolates**

The isolate selection includes 61 isolates comprising 13 serotypes and nine global pneumococcal sequence clusters (GPSC). The inner metadata ring denotes GPSC and the outer ring serotype.

**
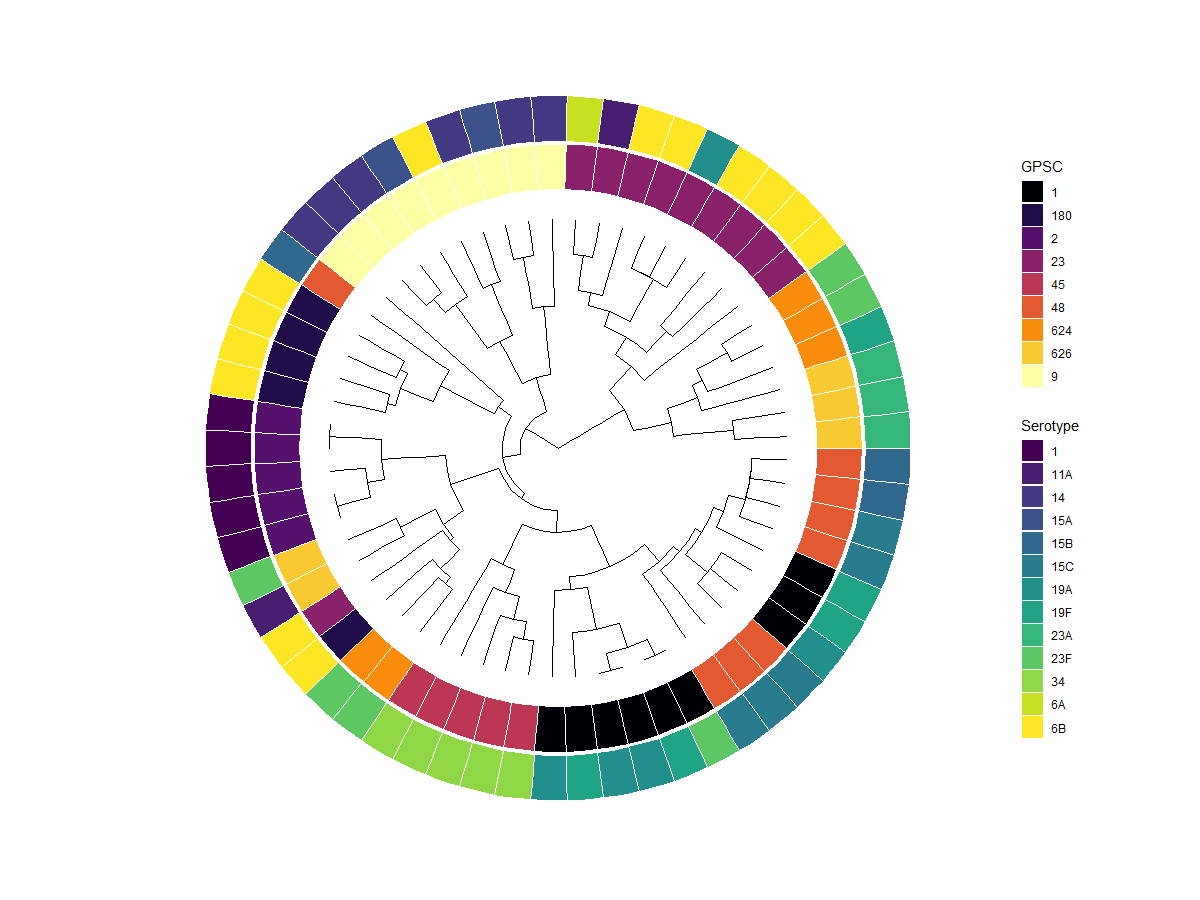
**
