# Supplementary material for: MALDI-TOF mass spectrometry for sub-typing of Streptococcus pneumoniae
Source: BMC Microbiol. 2020 Dec 1;20:367. doi: 10.1186/s12866-020-02052-7 (PMC7709296; doi:10.1186/s12866-020-02052-7)
Supplement: Supplementary file 4 — Additional file 4 Characteristics of Random Forest and CART algorithms for serotype- and genotype-associated MALDI-TOF mass spectra from 61 Streptococcus pneumoniae isolates from nine dominant global pneumococcal sequence clusters (GPSC) comprised of 13 serotypes. [file 12866_2020_2052_MOESM4_ESM.docx]

**Characteristics of Random Forest and CART algorithms for serotype- and genotype-associated MALDI-TOF mass spectra from 61 *Streptococcus pneumoniae* isolates from nine dominant global pneumococcal sequence clusters (GPSC) comprised of 13 serotypes**

| **Data organisation** | **Class** | **Random Forest** | | | **CART** | | |
| --- | --- | --- | --- | --- | --- | --- | --- |
|  |  | **TP Rate*** | **FP Rate†** | **ROC‡ Area** | **TP Rate** | **FP Rate** | **ROC Area** |
| **Serotype** | 1 | 0.6 | 0.036 | 0.934 | 0.6 | 0.018 | 0.786 |
|  | 6A | 0 | 0 | 0.442 | 0 | 0 | 0.367 |
|  | 6B | 0.462 | 0.354 | 0.645 | 0.846 | 0.146 | 0.836 |
|  | 11A | 0 | 0.017 | 0.364 | 0 | 0 | 0.237 |
|  | 14 | 0.167 | 0.091 | 0.78 | 0.167 | 0.055 | 0.762 |
|  | 15A | 0 | 0.017 | 0.373 | 0 | 0 | 0.381 |
|  | 15B | 0 | 0 | 0.563 | 0 | 0.017 | 0.773 |
|  | 15C | 0 | 0.036 | 0.905 | 0.4 | 0.071 | 0.663 |
|  | 19F | 0.2 | 0.054 | 0.616 | 0.2 | 0.179 | 0.655 |
|  | 19A | 0 | 0.071 | 0.57 | 0 | 0.018 | 0.53 |
|  | 23F | 0 | 0.182 | 0.341 | 0.5 | 0.109 | 0.767 |
|  | 23A | 0 | 0.052 | 0.678 | 0 | 0.034 | 0.911 |
|  | 34 | 0.4 | 0 | 0.836 | 0.8 | 0.018 | 0.891 |
|  | **Weighted average** | **0.213** | **0.122** | **0.657** | **0.41** | **0.075** | **0.727** |
| **Genotype** | GPSC1 | 0.778 | 0.231 | 0.835 | 1 | 0 | 1 |
|  | GPSC2 | 0.4 | 0.036 | 0.909 | 0.6 | 0.018 | 0.886 |
|  | GPSC9 | 0.444 | 0.019 | 0.888 | 0.889 | 0 | 0.933 |
|  | GPSC23 | 0.7 | 0.098 | 0.84 | 1 | 0 | 1 |
|  | GPSC45 | 0.2 | 0 | 0.957 | 0.4 | 0 | 0.795 |
|  | GPSC48 | 0.5 | 0.151 | 0.785 | 0.875 | 0.075 | 0.908 |
|  | GPSC180 | 0 | 0 | 0.745 | 0.8 | 0.036 | 0.866 |
|  | GPSC624 | 0.5 | 0.091 | 0.827 | 0.5 | 0.091 | 0.702 |
|  | GPSC626 | 0 | 0 | 0.509 | 0.25 | 0.035 | 0.822 |
|  | **Weighted average** | **0.459** | **0.085** | **0.824** | **0.77** | **0.026** | **0.9** |

*True positive, †False positive, ‡Receiver operating characteristic
