# Supplementary material for: MALDI-TOF mass spectrometry for sub-typing of Streptococcus pneumoniae
Source: BMC Microbiol. 2020 Dec 1;20:367. doi: 10.1186/s12866-020-02052-7 (PMC7709296; doi:10.1186/s12866-020-02052-7)
Supplement: Supplementary file 5 — Additional file 5. Discriminant peak list / matrix derived from 69 pneumococcal isolates, comprising tree dominant serotype-genotype pairs. [file 12866_2020_2052_MOESM5_ESM.docx]

**Discriminant peak list / matrix derived from 69 pneumococcal isolates, comprising three dominant serotype-genotype pairs.** The number in each cell summarises the proportion of isolates of the serotype with the corresponding mass peak.

| **Peak (m/z)** | **FDR* q-value** | **Serotype** | | |
| --- | --- | --- | --- | --- |
|  |  | **6B** | **19F** | **23F** |
| 2015.51 | 0.02443 | 0.00 | 0.40 | 0.07 |
| 3320.17 | 0.00000 | 0.00 | 0.95 | 0.21 |
| 3326.31 | 0.03458 | 0.40 | 0.00 | 0.10 |
| 3334.33 | 0.00000 | 0.60 | 0.00 | 0.31 |
| 3452.26 | 0.03613 | 0.40 | 0.10 | 0.03 |
| 3459.08 | 0.00000 | 0.00 | 0.00 | 0.59 |
| 3467.62 | 0.03807 | 0.30 | 0.10 | 0.59 |
| 3805.92 | 0.04391 | 0.00 | 0.25 | 0.00 |
| 3876.75 | 0.00351 | 0.00 | 0.40 | 0.00 |
| 4197.85 | 0.00351 | 0.65 | 0.10 | 0.72 |
| 4213.26 | 0.00000 | 0.00 | 0.80 | 0.00 |
| 4222.74 | 0.02443 | 0.00 | 0.30 | 0.00 |
| 4969.78 | 0.02186 | 0.00 | 0.00 | 0.31 |
| 4972.48 | 0.00000 | 0.05 | 0.80 | 0.66 |
| 5001.62 | 0.01984 | 0.40 | 0.00 | 0.03 |
| 5540.92 | 0.03807 | 0.00 | 0.00 | 0.28 |
| 5975.21 | 0.03613 | 0.10 | 0.50 | 0.10 |
| 6509.32 | 0.00000 | 0.45 | 0.75 | 0.00 |
| 6617.46 | 0.03807 | 0.35 | 0.05 | 0.55 |
| 6628.89 | 0.00351 | 0.50 | 0.00 | 0.17 |
| 6642.45 | 0.00000 | 0.00 | 0.85 | 0.28 |
| 6662.84 | 0.00000 | 0.00 | 0.70 | 0.21 |
| 6670.51 | 0.02443 | 0.50 | 0.00 | 0.31 |
| 6672.56 | 0.03458 | 0.50 | 0.00 | 0.34 |
| 6694.68 | 0.02443 | 0.50 | 0.00 | 0.31 |
| 6906.73 | 0.02810 | 0.35 | 0.15 | 0.00 |
| 6909.85 | 0.00000 | 0.60 | 0.55 | 0.03 |
| 6920.45 | 0.00000 | 0.00 | 0.05 | 0.72 |
| 8396.35 | 0.00000 | 0.70 | 0.05 | 0.72 |
| 8425.59 | 0.00000 | 0.00 | 0.60 | 0.00 |
| 8427.96 | 0.02443 | 0.00 | 0.30 | 0.00 |
| 8820.73 | 0.00000 | 0.00 | 0.00 | 0.45 |

*False discovery rate
