# Supplementary material for: MALDI-TOF mass spectrometry for sub-typing of Streptococcus pneumoniae
Source: BMC Microbiol. 2020 Dec 1;20:367. doi: 10.1186/s12866-020-02052-7 (PMC7709296; doi:10.1186/s12866-020-02052-7)
Supplement: Supplementary file 6 — Additional file 6 Characteristics of Random Forest and CART algorithms for serotype- and genotype-associated MALDI-TOF mass spectra from 69 Streptococcus pneumoniae isolates from three serotype - global pneumococcal sequence cluster (GPSC) pairs. [file 12866_2020_2052_MOESM6_ESM.docx]

**Characteristics of Random Forest and CART algorithms for serotype- and genotype-associated MALDI-ToF mass spectra from 69 *Streptococcus pneumoniae* isolates from three serotype - global pneumococcal sequence cluster (GPSC) pairs**

| **Data organisation** | **Class** | **Random Forest** | | | **CART** | | |
| --- | --- | --- | --- | --- | --- | --- | --- |
|  |  | **TP Rate*** | **FP Rate†** | **ROC‡ Area** | **TP Rate** | **FP Rate** | **ROC Area** |
| **Serotype** | 6B | 0.65 | 0.102 | 0.882 | 0.95 | 0.061 | 0.954 |
|  | 19F | 0.9 | 0.041 | 0.972 | 0.95 | 1 | 0.999 |
|  | 23F | 0.862 | 0.15 | 0.906 | 0.931 | 0.025 | 0.939 |
|  | **Weighted average** | **0.812** | **0.104** | **0.918** | **0.942** | **0.942** | **0.961** |
| **Genotype** | GPSC1 | 0.9 | 0.041 | 0.979 | 0.95 | 0 | 0.975 |
|  | GPSC23 | 0.6 | 0.122 | 0.876 | 1 | 0.041 | 0.971 |
|  | GPSC624 | 0.793 | 0.2 | 0.893 | 0.966 | 0 | 0.975 |
|  | **Weighted average** | **0.768** | **0.131** | **0.913** | **0.971** | **0.012** | **0.974** |

*True positive, †False positive, ‡Receiver operating characteristic
