# Supplementary material for: MALDI-TOF mass spectrometry for sub-typing of Streptococcus pneumoniae
Source: BMC Microbiol. 2020 Dec 1;20:367. doi: 10.1186/s12866-020-02052-7 (PMC7709296; doi:10.1186/s12866-020-02052-7)
Supplement: Supplementary file 7 — Additional file 7. A cluster dendrogram of serotype-organised MALDI-TOF mass spectrum data for the three dominant pneumococcal serotype-genotype pairs. [file 12866_2020_2052_MOESM7_ESM.docx]

**A cluster dendrogram of serotype-organised MALDI-TOF mass spectrum data for the three dominant pneumococcal serotype-genotype pairs**

The isolate selection comprises 69 pneumococcal isolates. The inner metadata ring denotes global pneumococcal sequence type (GPSC) and the outer ring serotype.

**
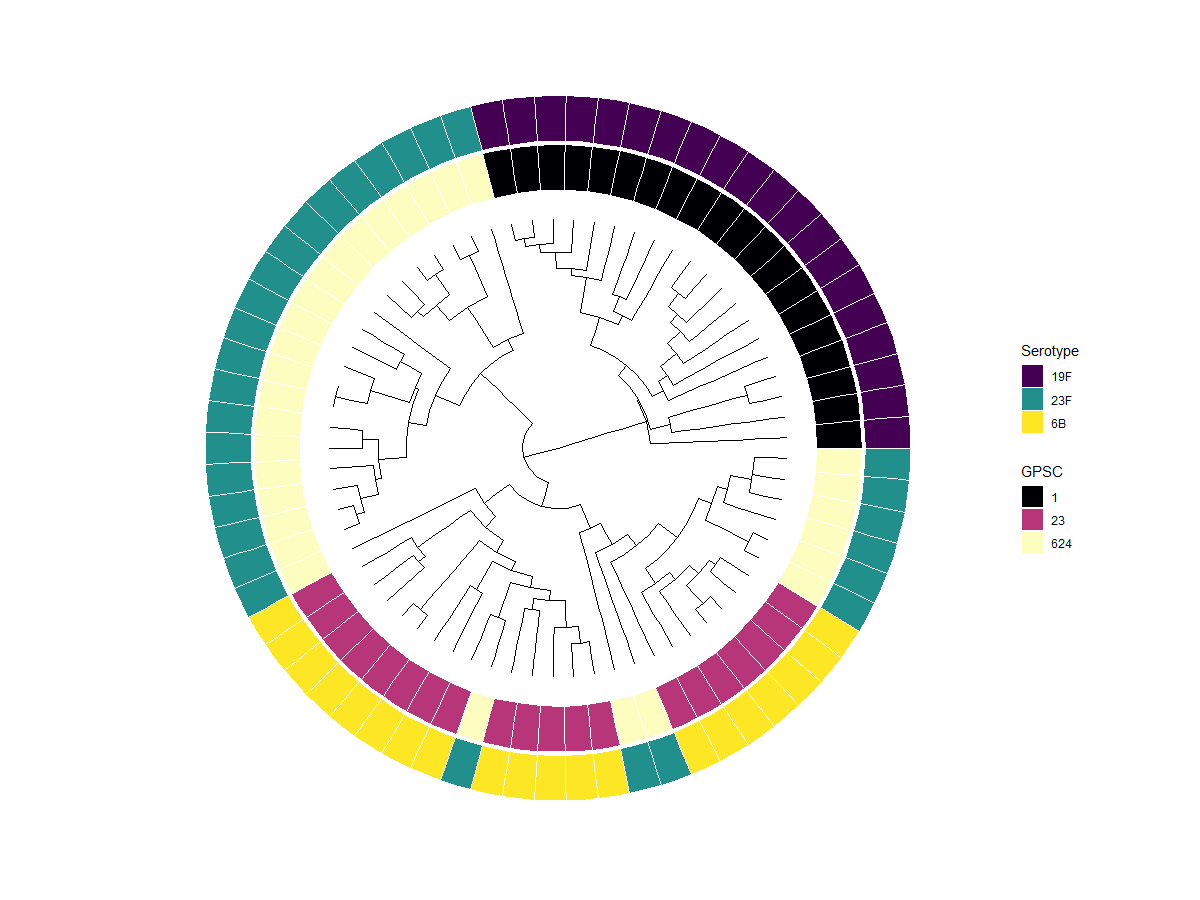
**
